# Sham Surgery and Inter-Individual Heterogeneity Are Major Determinants of Monocyte Subset Kinetics in a Mouse Model of Myocardial Infarction

**DOI:** 10.1371/journal.pone.0098456

**Published:** 2014-06-03

**Authors:** Jedrzej Hoffmann, Manuel Ospelt, Christian Troidl, Sandra Voss, Christoph Liebetrau, Won-Keun Kim, Andreas Rolf, Astrid Wietelmann, Thomas Braun, Kerstin Troidl, Sakthivel Sadayappan, David Barefield, Christian Hamm, Holger Nef, Helge Möllmann

**Affiliations:** 1 Department of Cardiology, Kerckhoff Heart and Thorax Center, Bad Nauheim, Germany; 2 Department of Cardiology, University of Giessen, Medical Clinic I, Giessen, Germany; 3 Max Planck Institute for Heart and Lung Research, Bad Nauheim, Germany; 4 Department of Cell and Molecular Physiology, Health Sciences Division, Loyola University Chicago, Maywood, Illinois, United States of America; Albert Einstein College of Medicine, United States of America

## Abstract

**Aims:**

Mouse models of myocardial infarction (MI) are commonly used to explore the pathophysiological role of the monocytic response in myocardial injury and to develop translational strategies. However, no study thus far has examined the potential impact of inter-individual variability and sham surgical procedures on monocyte subset kinetics after experimental MI in mice. Our goal was to investigate determinants of systemic myeloid cell subset shifts in C57BL/6 mice following MI by developing a protocol for sequential extensive flow cytometry (FCM).

**Methods and Results:**

Following cross-sectional multiplex FCM analysis we provide for the first time a detailed description of absolute quantities, relative subset composition, and biological variability of circulating classical, intermediate, and non-classical monocyte subsets in C57BL/6 mice. By using intra-individual longitudinal measurements after MI induction, a time course of classical and non-classical monocytosis was recorded. This approach disclosed a significant reduction of monocyte subset dispersion across all investigated time points following MI. We found that in the current invasive model of chronic MI the global pattern of systemic monocyte kinetics is mainly determined by a nonspecific inflammatory response to sham surgery and not by the extent of myocardial injury.

**Conclusions:**

Application of sequential multiplexed FCM may help to reduce the impact of biological variability in C57BL/6 mice. Furthermore, the confounding influence of sham surgical procedures should always be considered when measuring monocyte subset kinetics in a murine model of MI.

## Introduction

Monocytes are key components of the innate host immune defense; however, the imbalance of monocytic responses with resulting prolonged inflammation may aggravate disease. Different human monocyte subsets have been postulated to play distinct roles during infection as well as in sterile inflammatory disorders, including atherosclerosis, acute myocardial infarction (MI), congestive heart failure, multiple sclerosis, and cancer [1], [2]. Specific phenotypic and functional similarities between the main mouse and human monocyte subpopulations, including so-called “classical” (Ly6C^hi^ CD43^low^ in mice and CD14^high^CD16^neg^ in humans), “non-classical” (Ly6C^low^CD43^high^ and CD14^high^CD16^neg^, respectively), and “intermediate” subsets, suggest that the role of mouse monocytes in murine models of disease might parallel the role of human monocytes in corresponding human disease [3]–[5]. Mouse models of MI are commonly used to explore the basic pathophysiological and prognostic role of the monocytic response following myocardial injury. The occurrence of blood monocytosis was correlated with the extent of myocardial injury and with impaired myocardial salvage in patients following acute MI, and increased numbers of circulating monocytes were also found to predict impaired infarct healing and plaque progression in atherosclerotic mice [6], [7]. Accordingly, abrogating blood monocytosis to counteract the progression of atherosclerotic disease or to improve infarct healing in mice may emerge as a promising future approach to the treatment of ischemic heart diseases in humans [8], [9]. However, to develop reliable translational strategies, it is necessary to determine the specificity of the post-infarction hematological response in a mouse model of MI. In particular, no study thus far has examined the impact of biological inter-individual variability and invasive sham procedures on monocyte subset kinetics and development of monocytosis after experimental MI in mice.

In this study, we use low-volume, high-throughput, multi-parameter flow cytometry to assess heterogeneity and biological variability of the distinct circulating granulocyte and monocyte/macrophage subsets in wild-type C57BL/6 mice and to investigate determinants of systemic myeloid cell subset shifts in these mice following MI.

## Methods

### Mice

All animal experiments were performed according to the guidelines published by the Society for Laboratory Animal Science and were in compliance with the Guide for the Care and Use of Laboratory Animals published by the US National Institutes of Health (NIH publication No. 85–23, revised 1996) and were approved by the appropriate local authorities on animal care (Regierungspräsidium Darmstadt, Hessen, Germany). Nine- to eleven-week-old C57BL/6 wild-type mice (all male) were analyzed in this study. Animals were housed in temperature-controlled facilities on a 12-h light/dark cycle.

### Mouse model of myocardial infarction

All surgical procedures were performed as previously described [10]. Mice were sedated by placing them into an isoflurane induction chamber containing an isoflurane/air mixture (5% Forene) and were monitored until recumbent. Animals were then endotracheally intubated and mechanically ventilated with an isoflurane/air mixture (2,5% Forene) through a rodent ventilator (MiniVent, Sachs, March-Hugstetten, Germany). A left lateral thoracotomy was performed at the fourth intercostal space. A 7–0 prolene ligature was placed around the left anterior descending artery just below the atrioventricular border. Ischemia was evident from ECG alterations and discoloration of the infarct artery-related area. The muscle and skin layers were closed with sutures. The animals were weaned from the respirator and extubated. Sham-operated animals (MI control) were subjected to similar surgery, passing the thread without ligating the artery. The animals were sacrificed 2, 3, 5, or 7 days after induction of MI (n = 18–23 per time point). For the time-course study, mice were sequentially analyzed within 21 days following MI induction (n = 15) and sham procedure (n = 7). In addition, sequential blood sampling from non-operated mice was performed for 5 days (sampling control, n = 4).

### Blood sampling techniques

Blood sampling from the orbital sinus was performed for terminal blood collection by trained personnel, as described previously [11]. Briefly, wild-type (n = 180) or post-MI mice were anesthetized with a mixture of ketamine (100 mg/kg body weight) and xylazine (6 mg/kg body weight) intraperitoneally and one orbital sinus was punctured using a glass pipette. Blood (0.8–1.0 ml) was collected into EDTA tubes and 20 or 50 µl were immediately transferred into BD TruCount tubes using reverse pipetting. For serial sampling, 20 µl of blood were obtained by a trained investigator via bleeding from the submandibular vein using a lancet (without anesthesia) as described previously [12] and immediately transferred into BD TruCount tubes for antibody staining.

### Staining for flow cytometry (FCM) and data acquisition

Circulating blood leukocyte subpopulations in mice were assessed using a polychromatic fluorescent bead-based flow cytometry assay (BD TruCount, BD Biosciences, San Jose, CA, USA). Peripheral blood (PB) samples (20 or 50 µl) were stained for 20 min at room temperature with a panel of monoclonal antibodies: anti-CD43-FITC (BioLegend); anti-Ly6G-PE, anti-CD90.2-PE, anti-B220-PE, anti-CD49b-PE (VLA-2 alpha chain), anti-NK1.1-PE (BD Biosciences); anti-MHC-II-(Iab)-PE/Cy7 (BD Biosciences); anti-F4/80-APC, anti-CD11b-AlexaFluor700 (BD Biosciences); anti-Ly6C-PacificBlue (BioLegend). All antibody conjugates were carefully titrated prior to use. Red blood cells were lysed in tubes by adding 450 µl (or 250 µl for 20 µl PB) ammonium chloride-based lysis buffer (BD PharmLyse, BD Biosciences, San Jose) and samples were incubated for another 15 min at room temperature. Data acquisition was performed on a BD LSR II flow cytometer using FACSDiva software (BD). The acquisition flow rate was optimized for different blood sample volumes in the preliminary experiments. At least 30,000 leukocyte events and 3000 TruCount beads were acquired in a single measurement (the threshold was set on SSC and AlexaFluor700 was adjusted for optimal bead visualization).

### Flow cytometer setup

The Becton Dickinson LSR II flow cytometer was equipped with 3 spatially and time-delayed lasers: blue (488 nm), UV (355 nm), and red (638 nm). Prior to any analysis the instrument was checked for correct laser delay settings and fluidics stability by calibrating the results with cytometer tracking and setup beads using an acceptable tolerance of +/− 10 volts (Becton Dickinson) and single-peak Spherotech Ultra Rainbow beads. At the time of experimental set up the PMT voltages were adjusted for each fluorochrome with the objective of optimizing the signal-to-noise ratio, based on single stained blood samples. Spectral overlap between different channels was calculated automatically by the FACSDiva software after measuring single-color compensation controls (blood samples obtained from a healthy wild-type C57BL/6 mouse). Due to the relatively low number of APC-positive events following anti-F4/80 staining, the anti-CD115 APC conjugate was used for the APC compensation control instead.

### Flow cytometry data analysis

The analysis of acquired data was performed using FlowJo software (Version 9.4.1 for Macintosh, Tree Star, Inc.). Two-dimensional plots (pseudo-color, classical dot-plot or zebra) were created using bi-exponential transformation, and sequential gating of monocytes was performed according to the model of monocyte differentiation as proposed by Ziegler-Heitbrock et al. [13]. Median channel values for pertinent fluorescence detectors were recorded for particular cell subsets as expressed by “median fluorescence intensity” (MFI). A software-integrated Boolean gating tool was used to generate combined gates for continuous cell populations (intermediate monocytes). Absolute cell counts per µl PB were calculated according to manufacturer's protocol (BD Bioscience).

### Immunohistochemical analysis

Serial cryosections (6 µm) were prepared of the whole myocardium, including the infarct, peri-infarct, and remote areas. Samples were transferred to silane-coated glass-slides, air-dried, and fixed in paraformaldehyde. All sections were incubated with the specific fluorochrome-conjugated antisera: anti-CD68-AlexaFluor647 (MCA1957A647T, AbD Serotec, Puchheim, Germany) and anti-MRC1-AlexaFluor488 (clone MR5D3, BioLegend, London, UK). Antibody dilution was 1∶100. Nuclei were counterstained with DAPI (1∶1000, Invitrogen, Carlsbad, CA, USA) for 20 minutes at room temperature. Sections were viewed in a Leica TCS SP5 laser scanning confocal microscope. To quantify the number of inflammatory cells present in the infarcted tissue, 6 representative high-power images (×400) per animal were analysed with image analysis software (Image J 1.44p, NIH, Bethesda, MD, USA). Cell density was expressed as cells/mm^2^.

### MRI analysis

Serial cardiac magnetic resonance imaging (MRI) measurements were performed under isoflurane anesthesia on a 7.0 T Bruker Pharmascan equipped with a 300 mT/m gradient system using a custom-built circularly polarized birdcage resonator and the IntraGate self-gating tool (Bruker, Ettlingen, Germany). The measurement is based on the gradient echo method (repetition time = 6.2 ms; echo time = 1.6 ms; field of view = 2.20×2.20 cm; slice thickness = 1.0 mm; matrix = 128×128; repetitions = 100). The imaging plane was localized using scout images showing the 2- and 4-chamber view of the heart; this was followed by acquisition in short-axis view, orthogonal on the septum in both scouts. Multiple contiguous short-axis slices consisting of 7 to 10 slices were acquired for complete coverage of the left and right ventricle. MRI data were analyzed using the QMass MR software (version 7.1; Medis, Leiden, Netherlands). Functional parameters were assessed before MI and on days 1, 7, and 21 following induction of MI. The infarct was characterized as the area of akinesis and the infarct size measurements were performed as described previously [14], [15].

### Statistical analysis

In the text, data are reported as mean±SE. Comparison of the means was performed by ANOVA followed by Tukey's post-hoc test. Comparison of 2 groups was calculated using an unpaired t-test if normal probability plots (P-P plots) demonstrated approximate normality. Correlation analyses were performed with the use of Spearman rank coefficient. All statistical tests were performed using GraphPad Prism version 5 for Macintosh (www.graphpad.com).

## Results

### In-depth FCM analysis is adaptable for sequential blood sampling in mice

**Figure 1:**
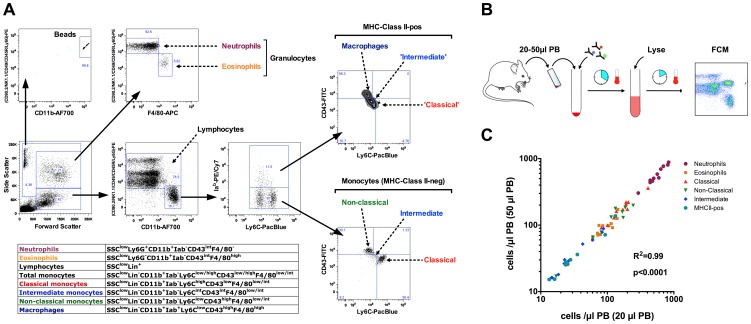
Polychromatic FCM is adaptable for low-volume measurements in mice. (**A**) Basic FCM gating strategy and phenotypic definitions for granulocyte and monocyte subsets as used in the current study. Monocytes (Lin**^−^**CD11b**^−^**Iab**^−^**) and MHC(Iab)^+^ cells were separated into main subsets based on the Ly6C/CD43 expression properties. (**B**) FCM immunobead-based assay for rapid leukocyte quantification in mice. (**C**) Comparison of assay-standardized (50 µl) and volume-reduced (20 µl) blood samples (n = 10 mice) show high correlation across the entire cell frequency range for all measured myeloid subsets.

We have refined a polychromatic flow cytometry protocol that allows for the direct high-throughput quantification of peripheral blood monocyte and granulocyte subsets in mice. By applying an extensive panel of FCM antibodies to a single-platform, lyse-no-wash, fluorescent bead-based assay, we were able to keep the pre-acquisition sample processing time under 40 minutes. This method optimally preserved the light-scattering properties of the murine leukocytes. Sufficient erythrocyte lysis allowed for light scatter-triggered collection of meaningful data. The sequential gating strategy used in the present study is shown in Figure 1A. The following myeloid cell subsets were simultaneously distinguished and quantified based on their immunophenotypical and light-scattering properties: neutrophil granulocytes (SSC^high^Lin^pos^CD11b^pos^Ly6G^pos^F4/80^neg^), eosinophil granulocytes (SSC^high^Lin^neg^CD11b^pos^Ly6G^neg^F4/80^pos^), classical monocytes (SSC^low^Lin^neg^CD11b^pos^Lin^neg^MHC-II^neg^CD43^low^Ly6C^high^), non-classical monocytes (SSC^low^Lin^neg^CD11b^pos^Lin^neg^CD43^high^Ly6C^low^), intermediate monocytes (SSC^low^Lin^neg^CD11b^pos^Lin^neg^Ly6C^int^CD43^int^) and activated monocytes/macrophages (SSC^low^Lin^neg^CD11b^pos^Lin^neg^MHCII^pos^Ly6C^low/int^CD43^int/high^) (Figure 1 A).

We further sought to adapt our protocol for non-lethal sequential blood sampling. According to current animal use guidelines, 1% of body weight can be safely drawn at daily intervals. Considering that the average body weight of the population of 180 C57BL/6 mice analyzed in our study was 25 g, the maximum daily blood sample volume should not exceed 20 µl (National Society for Laboratory Animal Science, 2009). On the other hand, as shown previously, using blood amounts below 20 µl could hinder the proper differentiation between monocyte subsets [16]. We therefore compared the concentration of monocyte and granulocyte subsets between assay-standardized (50 µl) and volume-reduced (20 µl) blood samples (Figure 1 B). There was an excellent correlation between both sample volumes, including absolute numbers and relative compositions of all cell populations measured (Figure 1 C).

### Variability of the multi-parameter FCM dataset

The variability of the multi-parameter immunophenotyping method was calculated following repetitive staining and measuring of blood sample duplicates from different animals (n = 12). The inter-assay coefficient of variation for absolute counts of monocyte and granulocyte subsets was 2.1% for classical, 2.1% for intermediate, and 4.6% for non-classical monocytes, and 3.8 and 5.1% for neutrophils and eosinophils, respectively.

### Myeloid cell subset frequencies are highly variable among wild-type C57BL/6 mice

**Figure 2:**
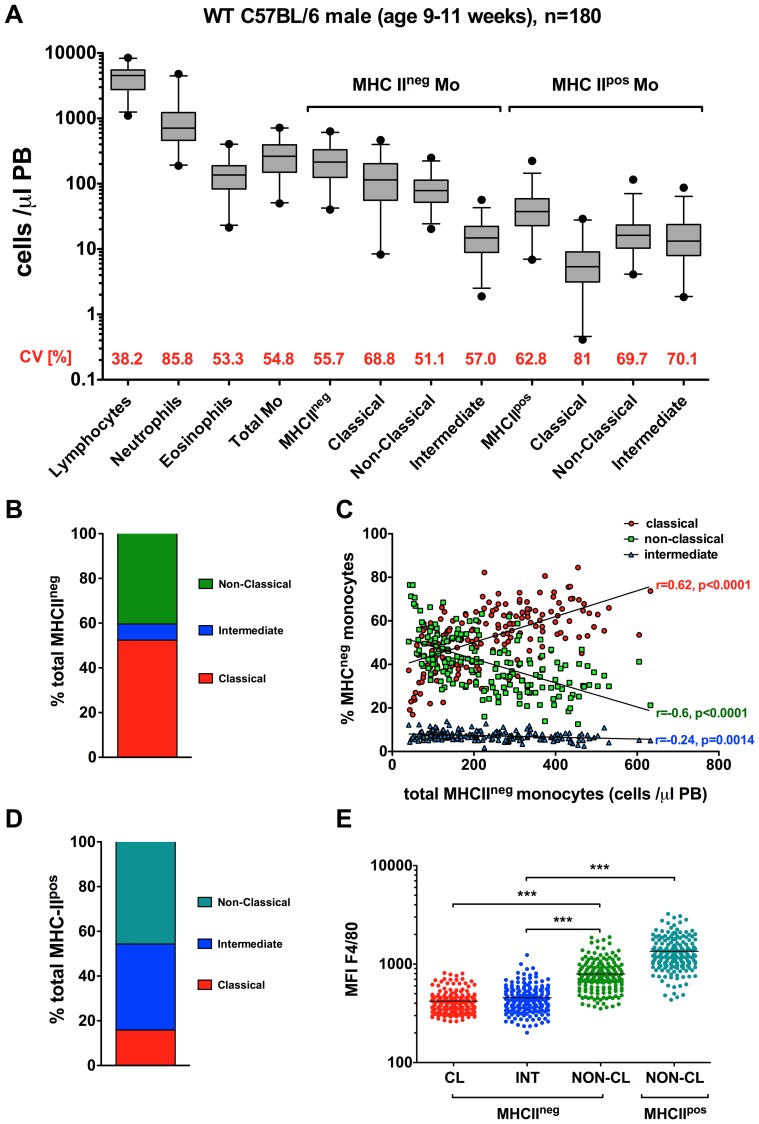
Inter-individual variability of circulating leukocyte subsets in C57BL/6 mice. (**A**) Cross-sectional cell frequency analysis of 180 gender- and age-matched wild-type (WT) mice. Box-and-whiskers plots of the absolute cell numbers. The box shows the 25^th^ to 75^th^ percentiles, and the line in the box indicates the median value. Horizontal bars outside the box indicate 10^th^ to 90^th^ percentiles and the circles indicate 1^st^ to 99^th^ percentiles. (CV – standard deviation/mean). (**B**) Frequencies of main monocyte subsets in WT mice as based on Ly6C/CD43 classification are displayed. (**C**) Occurrence of monocytosis in WT animals depends on shifts towards classical (Ly6C^hi^CD43^low^) phenotype. (**D**) Analysis of MHC-II-pos compartment reveals predominance of “non-classical” (Ly6C^low^CD43^high^) and “intermediate” phenotype. (**D**) Mean fluorescence intensity (MFI) for F4/80 in the major monocyte subsets in WT mice.

To investigate the pattern of leukocyte subset distribution in C57BL/6 mice we first analyzed whole blood samples collected from 180 matched animals. Remarkably, analysis of the absolute cell counts in the wild-type, apparently healthy, gender- and age-matched mice revealed a significant inter-individual variability in the frequencies of circulating leukocytes. The coefficient of variation (CV – standard deviation/mean) was used as an index of variability for distinct cell subsets. Accordingly, we identified neutrophil granulocytes (mean±SE 654.2±63.8 cells/µl; CV 85.8%), macrophages (18.7±1; 69.7%), and classical monocytes (135.6±7; 68.8%) as the most variable subsets, whereas eosinophils, non-classical monocytes, and lymphocytes showed the lowest inter-individual variability (144±5.7, 53.3%; 86.1±3.3, 51.1% and 4267±128.7, 38.2%, respectively) (Figure 2 A). In contrast, the relative composition of the major monocyte subsets was more conserved among tested animals. Accordingly, classical monocytes constituted 52.4±1%, non-classical 40.4±1%, and intermediate 7.2±0.2% of the entire monocyte population (SSC^low^CD11b^pos^Lin^neg^) (Figure 2 B). Of note, the absolute numbers of total blood monocytes were predominantly dependent on the proportion of the Ly6C^hi^ subset, indicating that the occurrence of Ly6C^hi^ monocytosis is a significant systemic immune variance in wild-type C57BL/6 mice (Figure 2 C).

Further analysis of the MHC-II^pos^ compartment, referred to as activated monocytes, revealed a predominance of the Ly6C^low/int^CD43^high/int^ cell phenotype (“non-classical”/”intermediate”) within this subset (Figure 2 D). We next compared the median fluorescence intensity (MFI) for F4/80, a prototypical macrophage antigen, in the 4 major subpopulations of monocytic cells [17]. The F4/80 MFI for MHCII^neg^ classical (421±9) and intermediate monocytes (455±12) did not differ, while MFI in MHCII^neg^ non-classical monocytes (794±23, p<0.0001 vs. classical) and MHCI^pos^ Ly6C^low^CD43^high^ (1350±23, p<0.0001 vs. all other subsets) was significantly higher than in MHCII^neg^ classical monocytes (Figure 2 E).

### Sham procedures influence systemic leukocyte shifts following MI in mice

**Figure 3:**
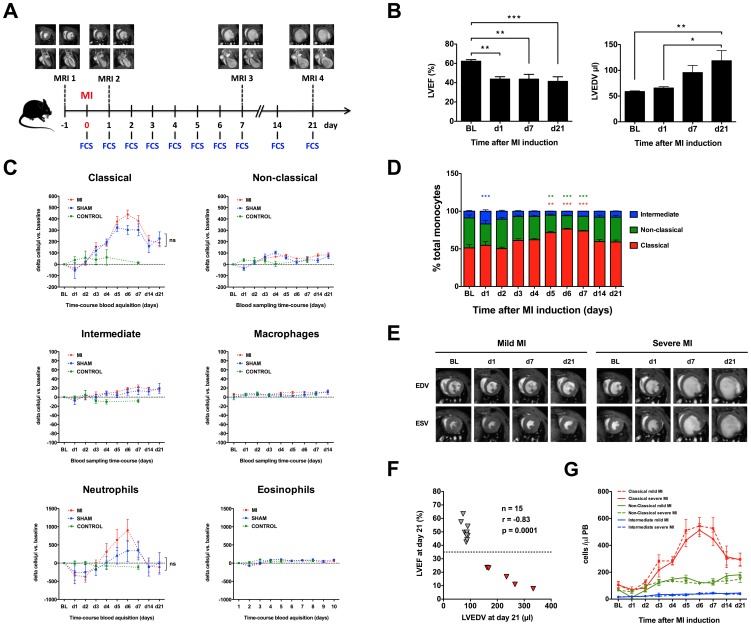
Sham surgery determines monocyte subset kinetics in a mouse model of MI. (**A**) Flow chart of the time-course study. (**B**) Mean ejection fraction (EF) and end-diastolic volume (EDV) obtained by sequential magnetic resonance imaging (MRI) in C57BL/6 mice (n = 15). Significance between time points was calculated by one-way ANOVA with Tukeys' post-hoc test: * p<0.05, ** p<0.01, *** p<0.001. (**C**) Sham-operated (n = 7) and MI mice displayed a similar intra-individual time course of circulating monocyte subsets. Calculated individual cell-delta data were used to display the intra-individual leukocyte subset kinetics and differences between MI, sham-operated and control (n = 4) groups. Significance between groups calculated by two-way ANOVA with Benferroni post-hoc test (MI vs. SHAM, ns - not significant). (**D**) Subset composition changes within the circulating MHCII^neg^ monocyte compartment following MI. (**E**) Example of MRI analysis with a short axis of hearts with mild and severe MI. EDV: end-diastolic volume, ESV: end-systolic volume. (**F**) Selection of mild versus severe MI based on the chronic impairment of the left ventricle ejection fraction (LVEF<35%) and increased LV dilatation (EDV) at day 21. (**G**) Monocyte time-course kinetics for both MI groups shows no correlation between development of blood monocytosis and the extent of myocardial injury.

In the second group of mice we looked at the time course of changes in the leukocyte inflammatory response after MI induction or the corresponding sham surgery. By using a currently recommended two-operator method of submandibular blood sampling a precise blood draw was assured while avoiding any excessive blood lost. For the baseline measurements, the intra-individual kinetics of circulating blood monocyte and granulocyte subsets was recorded on days 1, 2, 3, 4, 5, 6, 7, 14, and 21 following surgery. Functional cardiac parameters from recent to chronic myocardial infarction were recorded by sequential cardiac magnetic resonance imaging (Figure 3 A–B). Animals undergoing a successful coronary ligation procedure showed a ubiquitous drop in most of the cell subsets measured, in particular non-classical monocytes and eosinophil granulocytes, within the first 24 h after MI induction; this was followed by development of classical and non-classical monocytosis (Figure 3 C–D). Surprisingly, MI-induced and sham-operated animals displayed a very similar pattern of the circulating cell subset kinetics for all subsets analyzed. Significant effects of sequential blood collection were excluded by a time-course analysis of non-operated animals. These results show for the first time that common sham surgical procedures substantially confound measurements of circulating monocyte subsets in mice following MI. Accordingly, the magnitude of myeloid cell responses was independent of the extent of myocardial injury as no correlation was found between circulating cell subset levels and MRI-derived functional and morphological data (Figure 3 E–G).

### Cardiac monocytic infiltration is independent of peripheral monocytosis following MI in mice

To investigate the relationship between peripheral monocytosis and infiltrating monocytic subsets, we performed immunohistochemical staining and monocyte/macrophage quantification in sequentially preserved myocardial tissue specimens following MI. The results were correlated with peripheral blood monocyte subset numbers estimated by flow cytometry. We found no correlation between circulating and infiltrating cell numbers, which further proves that systemic monocytosis cannot be used as an indicator of a leukocyte-borne inflammation in the infarcted myocardium (Figure S1). To assess the effects of sham surgery on the myocardial inflammatory response we analyzed myocardial leukocyte subset infiltration in mice following MI and sham surgery (day 3) as well as in healthy, non-operated animals. Whereas infarcted animals showed apparent infiltration of monocyte/macrophages (CD68**^+^** cells, p<0.0001 vs. SHAM and CTRL) with a significant proportion of classical macrophages (MRC-1^neg^, p<0.0001 vs. SHAM and CTRL), no difference was detected between sham-operated and non-operated animals (Figures S2 and S3).

### Sequential FCM may reduce the impact of inter-individual variability in mice

**Figure 4:**
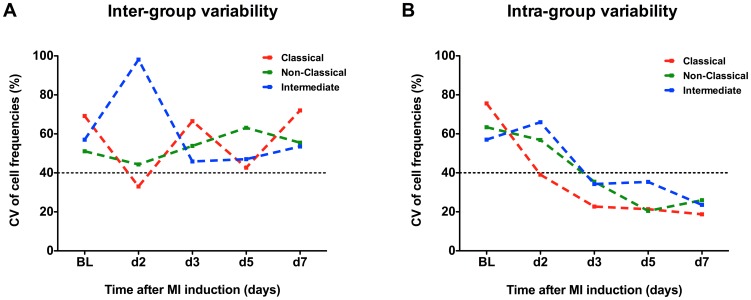
Sequential FCM may reduce the impact of inter-individual variability. Time course of the coefficient of variation for monocyte subset absolute cell numbers following MI within (**A**) inter-group (independently operated mice, n = 18–23/group) and (**B**) intra-group (sequential analysis, n = 8) experimental setups.

To examine effects of longitudinal acquisition on the inter-individual variability described above, we further calculated the time course of the coefficient of variation for monocyte subsets following MI within inter-group and intra-group experimental setups. Whereas animals from independent animal groups undergoing MI displayed high monocyte subset dispersion across all investigated time points following MI, significantly lower CV values were observed using within-group analysis. Here, the variance was still at its greatest at baseline (prior to MI induction) but was reduced by up to four-fold (classical monocytes) at day 7 post-MI. Figure 4 gives the spatial distribution of the inter- and intra-group variability of the main monocyte subsets.

## Discussion

Both the relative composition and absolute numbers of circulating myeloid cells are crucial parameters for quantifying the magnitude of a systemic inflammatory response. Applying new minimally invasive techniques for non-lethal monocyte subset measurements in mice may help to elucidate mechanisms regulating induction, progression, and resolution of the inflammatory response in different mouse models of human disease. Here we report on the successful design, validation, and large-cohort testing of a rapid and easily applicable protocol for extended phenotypic characterization and direct quantification of peripheral blood monocyte and granulocyte subsets in mice. In addition to its overall practicability, an important advantage of our approach is the gentle processing of cell samples assured by elimination of additional wash or centrifugation steps. This appears to be crucial for preserving the viability and optimal light-scattering properties of myeloid cell subsets, including granulocyte subpopulations. Selective staining of Ly6C and Ly6G ensured optimal differentiation between monocytes and neutrophil granulocytes. In addition to Ly6C, we included the anti-CD43 (leukosialin) antibody in the panel. This resulted in an optimal resolution of the monocyte population (SSC^low^Lin^neg^CD11b^pos^Iab^neg^Ly6C^low/high^) into classical, intermediate, and non-classical subsets. Furthermore, targeting of the prototypical macrophage marker F4/80 proved to be helpful as a non-redundant staining component in the present protocol. We demonstrated a significant gradient of staining intensity for F4/80 among monocytic cells. As F4/80 has been dubbed “monocyte maturation marker”, our analysis suggests that non-classical, but not intermediate, monocytes represent a higher differentiation stage in the monocytic lineage [3], [18]. This may indicate the transitional role of murine intermediate monocytes in the differentiation process from classical to non-classical subsets, as already postulated for human monocytes. However, as no functional or mechanistic link has been reported thus far for this subset in mice, one needs to be careful with speculations on the conversion of murine classical into non-classical monocytes. Moreover, in a very recent study, Jakubzick and colleagues proved in a series of elegant experiments that classical monocytes might retain their phenotypic properties without differentiation under non-inflammatory conditions [19]. Future studies need to provide more detailed phenotypic and functional characteristics and lineage commitment and to elucidate pathophysiological roles of intermediate monocytes in the mouse [20].

Following analysis of 180 perfectly age- and gender-matched animals, we provide for the first time a detailed description of the biological variation of circulating monocyte and granulocyte subsets in mice. Probably one of the most striking results of our study was a high inter-individual variability of myeloid cell frequencies within the C57BL/6 mouse strain. After ruling out technical artifacts (assay instability), we conclude that significant (temporary) shifts of inflammatory cell subsets may indeed occur in wild-type, apparently healthy animals. In examining this phenomenon, one should take circadian rhythms into account and also consider the potential impact of social and emotional stress in laboratory mice [21]. In particular, neutrophil granulocytes and classical monocytes, two prototypical “pro-inflammatory” circulating compartments of the innate immune response that can be quickly mobilized from their systemic reservoirs [22], [23] and rapidly react to environmental stimuli, may indeed represent the most variable immune component in mice. This hypothesis could explain the high CV values calculated for these two subsets. Furthermore, increasing monocyte frequencies strongly correlated with a shift towards the classical phenotype, providing further evidence for the occurrence of Ly6C^hi^ monocytosis as an important indicator of systemic ‘inflammatory’ status in wild-type animals. In contrast, some other cell subsets, including lymphocytes or eosinophil granulocytes, appeared to be less variable. However, as the current antibody panel setup did not allow for any differential analysis of the lymphocytic compartment, a potential dispersion of e.g. specific T-cell subsets might have remained underestimated within the present analysis.

Our study has yielded several important results. Firstly, we showed that in-depth analysis of complex immunophenotypic patterns within the myeloid compartment is possible in mice even when a minimal blood sample volume is used. We managed to establish a standardized single-platform protocol for a high-quality, minimal-volume, flow-cytometric analysis that enabled sequential identification and quantification of different monocyte and granulocyte populations. Secondly, we demonstrated for the first time, using a large collective of C57BL/6 mice, that even apparently healthy and perfectly matched wild-type animals of the most common laboratory strain can significantly differ with regard to circulating cell subset frequencies, and we showed that inter-individual variability can be a potential confounder in cross-sectional experimental setups. Furthermore, as an example of the practicability of our protocol, we identified intra-individual shifts of circulating myeloid cell subsets in a mouse model of chronic myocardial infarction. More importantly, our results exposed a hitherto underappreciated impact of sham procedures on the systemic monocyte subset response. Accordingly, the unmasking of any specific effects of myocardial ischemic injury may not be possible when shifts of circulating monocyte (e.g. Ly6Chi monocytosis) and granulocyte compartments are being analyzed. Our results are in line with some previous reports describing induction of a systemic cytokine response by the surgical procedures performed to induce murine myocardial infarction and reperfusion [24]. The use of minimally invasive “closed-chest” models of infarction that have been developed in the mouse could potentially help to reduce the confounding effects of the surgical procedure [24], [25]. Nevertheless, we conclude that, in terms of specificity of the cellular immune responses, myocardial infarction in mice should be considered to be a predominantly local phenomenon, at least when the currently most common model of invasive MI induction is being used. We were able show that the sham-associated peripheral mobilization of leukocyte subsets does not reflect the actual infarction-associated, leukocyte-borne cardiac inflammation. Hence, peripheral monocytosis in mouse models of MI cannot be interpreted as a specific phenomenon, which might be an important finding for future preclinical research on hematological epiphenomena of tissue injury. This is in contrast to acute MI in humans where specific systemic shifts of circulating leukocyte subsets are detectable and may be considered as a potential surrogate marker for cardiac injury [26], [27]. However, as our data refer only to a model of chronic infarction without the impact of atherosclerosis or subsequent reperfusion, we acknowledge that caution must be applied when drawing general conclusions about the significance of differences between mouse and human immune response to myocardial injury [6], [28].

Finally, we showed that application of minimal-volume FCM for longitudinal analyses might reduce the impact of inter-individual variability when the time course of blood-based inflammation is analyzed in mice. Thus, we postulate that intra-individual measurements represent the most feasible way to reduce bias arising from the large variation of leukocyte numbers. This approach would also serve to reduce the number of animals required for an experimental setup. This latter aspect would particularly apply when a hematological response is being studied in genetically modified mice such as the C57BL/6 strain, commonly used to generate transgenic founders, that are usually less available and more costly than other mouse models. However, future studies, possibly including both sexes and multiple-strain setups, should further elucidate the potential of intra-individual hematological measurements to increase the power of experiments with mice.

Multiple studies have already addressed monocyte and macrophage kinetics following myocardial injury in mice, including most recently sophisticated adoptive transfer-based experimental designs that have provided more insight into the mechanisms that regulate monocyte mobilization and recruitment into the infarcted myocardium [29]–[31]. Here we report some additional, previously mostly unappreciated hematological features of the murine myeloid compartment in steady state and inflammation. Our results suggest that minimal-volume sequential flow cytometry may help to reduce the impact of inter-individual variability and that confounding effects of surgical procedures should always be considered when measuring blood monocyte kinetics in mice.

## Supporting Information

Figure S1 **No significant correlation was found between peripheral blood monocyte subset numbers and myocardial monocyte/macrophage infiltration (CD68^+^ cells) at the different time points following MI induction in mice (intra-individual peripheral blood FCS analysis and immunohistochemical cell-density quantification of infarct area left-ventricular tissue specimens following MI, 6–8 animals/group).**
(TIFF)

Figure S2 **Immunohistochemical analysis of myocardial infiltration by monocytes/macrophages (CD68^+^ cells) in mice following myocardial infarction (MI day 3) or sham surgery (SHAM day 3) and in healthy, non-operated animals (CTRL).** Significance between time points was calculated by one-way ANOVA with Tukeys' post-hoc test (*** p<0.001, ns – not significant; 4 animals/group).
(TIFF)

Figure S3 **Infarcted animals showed apparent infiltration of monocyte/macrophages (CD68^+^ cells) with a significant proportion of ‘classical’ macrophages (MRC-1^neg^); no difference was detected between sham-operated and non-operated animals.** Significance between time points was calculated by one-way ANOVA with Tukeys' post-hoc test (*** p<0.001, ns – not significant; 4 animals/group).
(TIFF)
