# Supplementary figures and images for: Sham Surgery and Inter-Individual Heterogeneity Are Major Determinants of Monocyte Subset Kinetics in a Mouse Model of Myocardial Infarction
Source: PLoS One. 2014 Jun 3;9(6):e98456. doi: 10.1371/journal.pone.0098456 (PMC4043649; doi:10.1371/journal.pone.0098456)

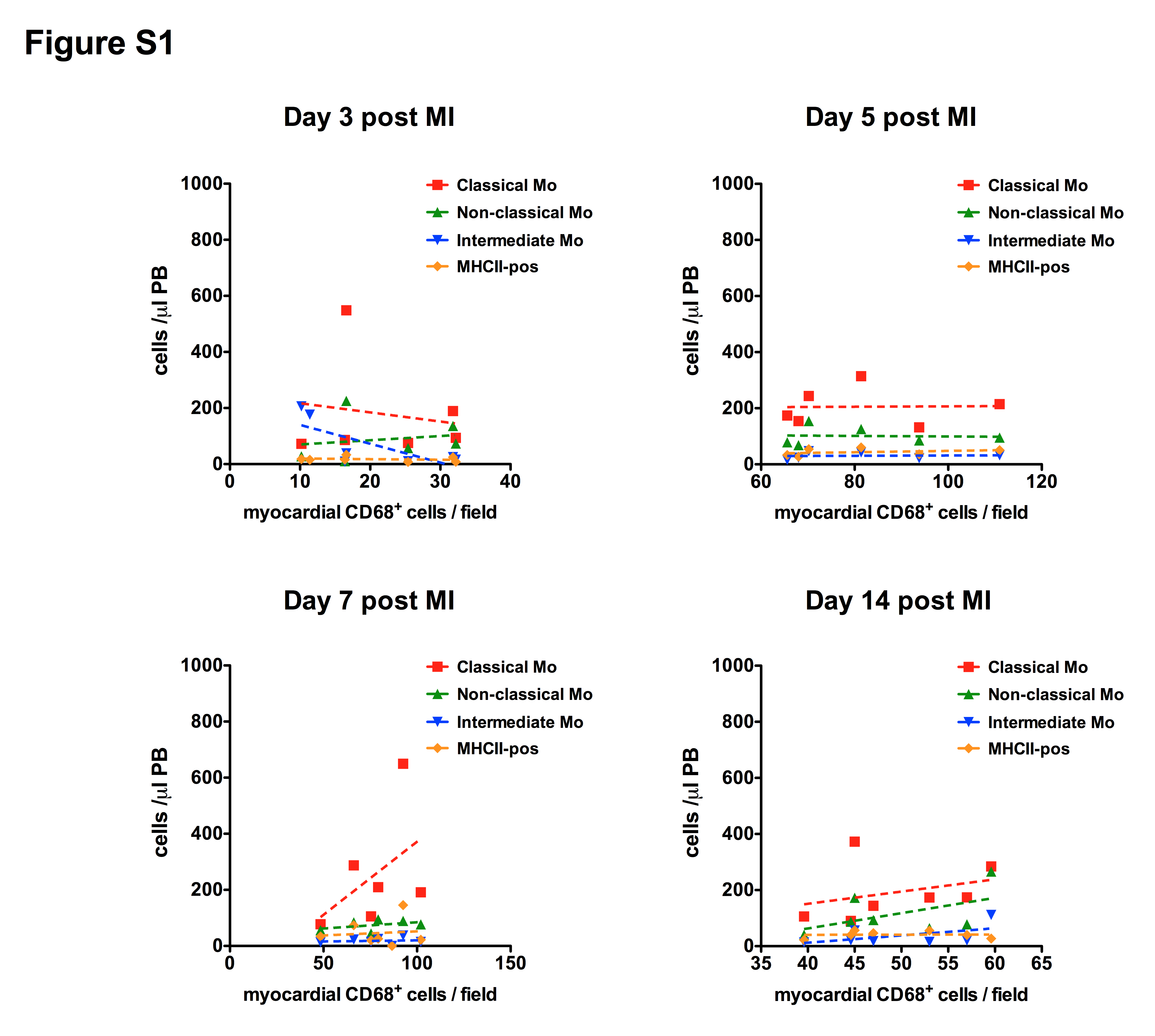

Supplement: Figure S1 — No significant correlation was found between peripheral blood monocyte subset numbers and myocardial monocyte/macrophage infiltration (CD68+ cells) at the different time points following MI induction in mice (intra-individual peripheral blood FCS analysis and immunohistochemical cell-density quantification of infarct area left-ventricular tissue specimens following MI, 6–8 animals/group). (TIFF) [file pone.0098456.s001.tif]

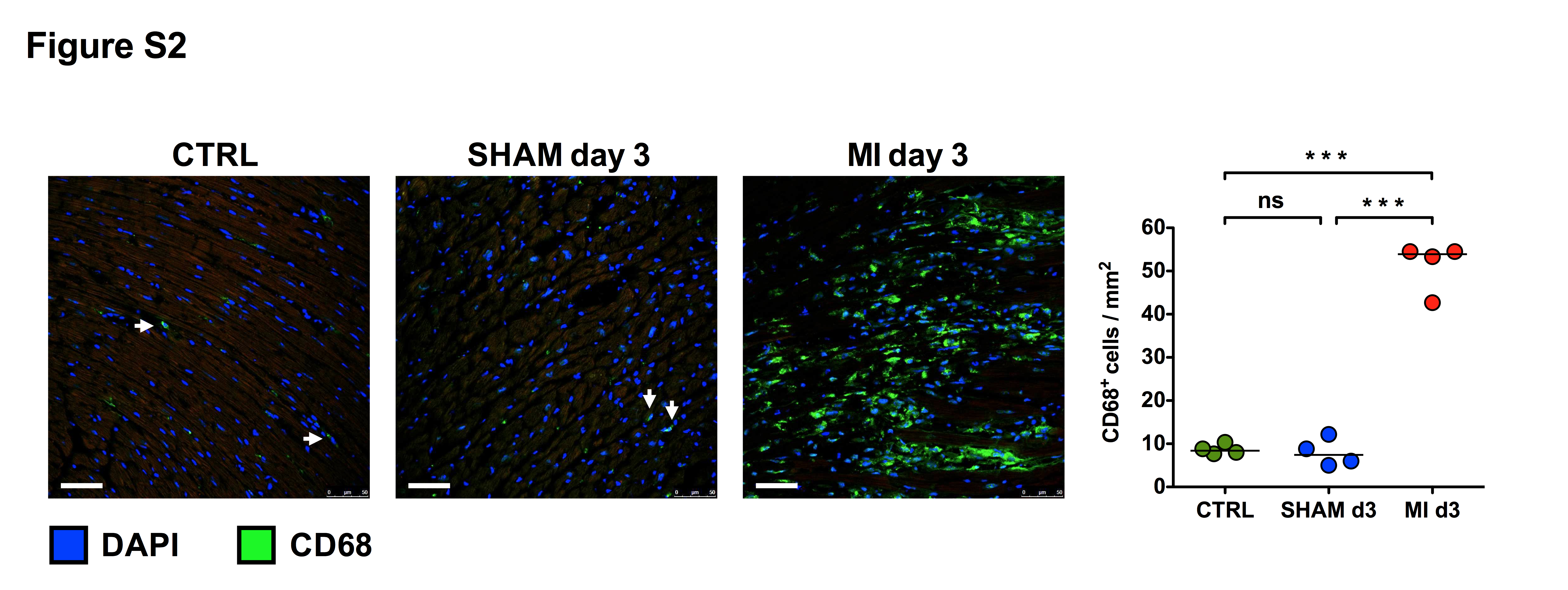

Supplement: Figure S2 — Immunohistochemical analysis of myocardial infiltration by monocytes/macrophages (CD68+ cells) in mice following myocardial infarction (MI day 3) or sham surgery (SHAM day 3) and in healthy, non-operated animals (CTRL). Significance between time points was calculated by one-way ANOVA with Tukeys' post-hoc test (*** p<0.001, ns – not significant; 4 animals/group). (TIFF) [file pone.0098456.s002.tif]

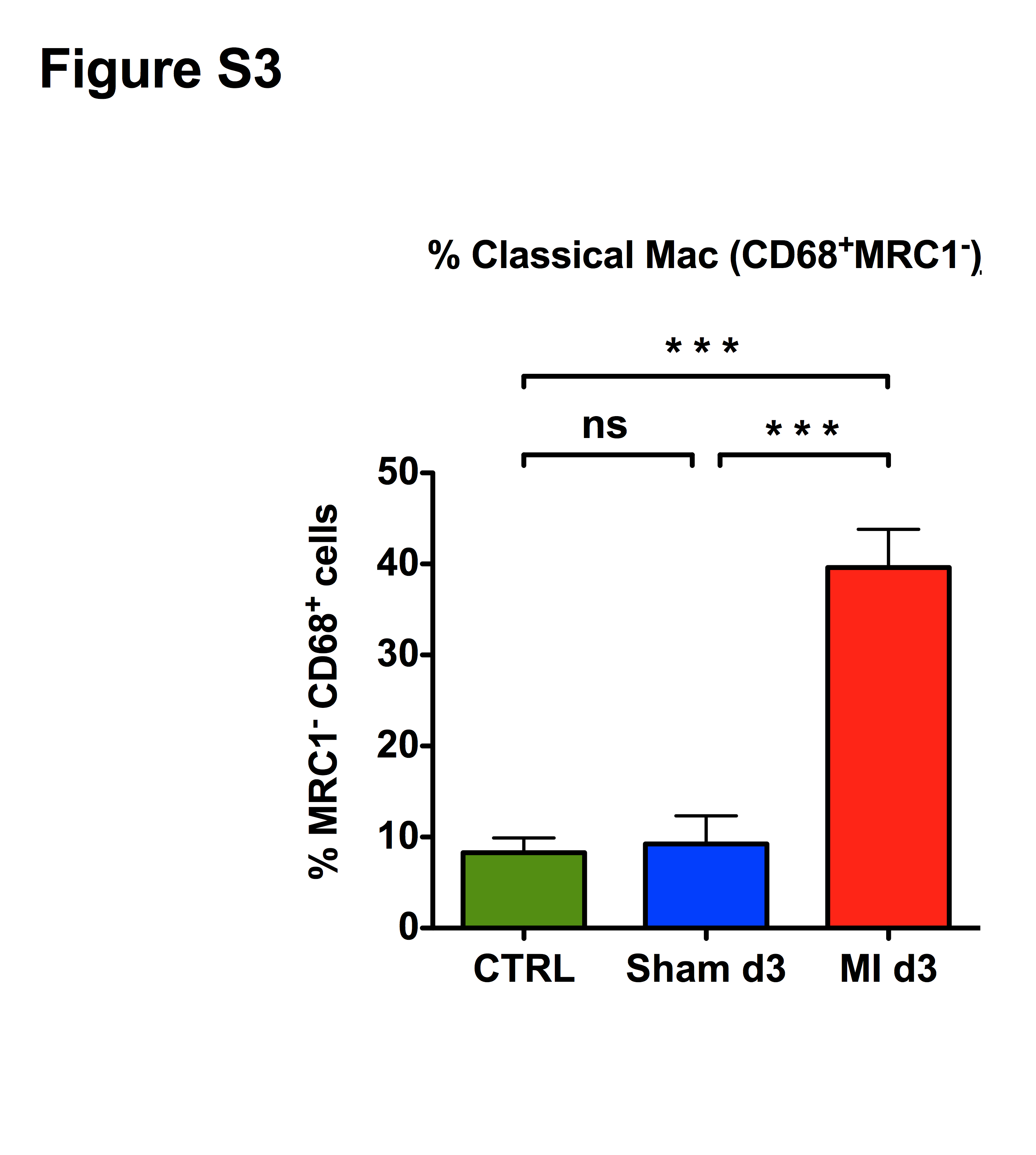

Supplement: Figure S3 — Infarcted animals showed apparent infiltration of monocyte/macrophages (CD68+ cells) with a significant proportion of ‘classical’ macrophages (MRC-1neg); no difference was detected between sham-operated and non-operated animals. Significance between time points was calculated by one-way ANOVA with Tukeys' post-hoc test (*** p<0.001, ns – not significant; 4 animals/group). (TIFF) [file pone.0098456.s003.tif]
